# Supplementation of Yoghurt with *Apilactobacillus kunkeei* Strain Ameliorates Non-Alcoholic Fatty Liver Disease in Rat Model

**DOI:** 10.3390/nu18030406

**Published:** 2026-01-26

**Authors:** Fouad M. F. Elshaghabee, Essam M. Hamad, Tarek A. Ebeid, Hashim S. Ibrahim, Waleed Al Abdulmonem

**Affiliations:** 1 Dairy Science Department, Faculty of Agriculture, Cairo University, Giza 12613, Egypt; elshaghabee@agr.cu.edu.eg; 2 Department of Food Science and Human Nutrition, College of Agriculture and Food, Qassim University, Buraidah 51452, Saudi Arabia; e.hamad@qu.edu.sa (E.M.H.); h.elsiddig@qu.edu.sa (H.S.I.); 3 Department of Animal and Poultry Production, College of Agriculture and Food, Qassim University, Buraidah 51452, Saudi Arabia; t.ebeid@qu.edu.sa; 4 Department of Pathology, College of Medicine, Qassim University, Buraidah 51452, Saudi Arabia

**Keywords:** high fructose diet, NAFLD, *Apilactobacillus kunkeei*, IL-6, IL-10, fecal ethanol

## Abstract

**Background/Objectives**: This study evaluated whether yoghurt containing *Apilactobacillus kunkeei* DSM 12361 protects rats against non-alcoholic fatty liver disease (NAFLD). We hypothesized that this fructophilic probiotic, with anti-inflammatory properties, may affect NAFLD progression by improving the gut microbiome, lowering intestinal ethanol production, and modulating inflammatory and metabolic pathways linked to hepatic fat accumulation. **Methods**: Wister rats were randomized into three groups; rats in the control group (HFrD) were fed a high-fructose (70%) diet while rats in experimental groups were fed the same diet mixed with 10% of yoghurt containing YC-180 starter culture (HFrD-Y) or yoghurt containing YC-180 and *Apilactobacillus kunkeei* DSM 12361 (HFrD-Y-A). **Results**: After six weeks of intervention, levels of plasma triglycerides, cholesterol, glucose, liver enzymes (ALT and AST), interleukin (IL)-6, fecal ethanol, *Enterobacteriaceae*, and hepatic index were significantly increased (*p* < 0.05) in the HFrD group as compared to rats in both experimental groups. Moreover, plasma levels of liver enzymes, lipid profile, glucose, and IL-6 were significantly lower (*p* < 0.05) in rats of the HFrD-Y-A group than those in the HFrD-Y group. Furthermore, plasma levels of IL-10 and fecal *Lactobacilli* and *Bifidobacteria* were significantly increased (*p* < 0.05) in the experimental groups when compared to rats in the control group. **Conclusions**: In sum, the obtained results indicated that yoghurt containing *Apilactobacillus kunkeei* could decrease the risk of non-alcoholic fatty liver disease (NAFLD) through (a) blocking the inflammation process associated with NAFLD, (b) enhancing the lipid profile, (c) lowering fecal ethanol, and (III) decreasing the levels of fecal *Enterobacteriaceae* in comparison with levels of fecal *Lactobacilli* and *Bifidobacteria* in rats. More research on molecular mechanisms of the potential effects of the *Apilactobacillus kunkeei* strain against NAFLD is still required.

## 1. Introduction

Non-alcoholic fatty liver disease (NAFLD) is a major metabolic disorder with significant health implications. It is one of the most common liver diseases globally, with an estimated prevalence of 32.4% among adults, with particularly high rates in the Middle East, especially the Gulf Countries [1,2,3,4,5]. The disease is associated with several factors, including obesity, type 2 diabetes, insulin resistance, and dietary patterns high in fructose, fats, and refined sugars. High fructose consumption contributes to fat accumulation, increased oxidative stress, and disruption of the gut–liver axis, highlighting the need for safe treatment strategies based on functional foods [6,7,8,9,10].

Research has shown that probiotics, and, in particular, lactic acid bacteria, have promising potential for preventing NAFLD by modifying the gut microbiota, supporting intestinal barrier function, and regulating metabolic processes [11,12,13]. Among these bacteria, *Apilactopacillus kunkeei*, a fructophilic bacterium found in honeybees, stands out for its unique metabolic, immunological, and safety properties [14,15]. Studies in mouse models have demonstrated its ability to reduce fructose-induced liver fat accumulation, restore gut microbiome balance, and improve intestinal mucosal permeability [16,17,18]. Further studies have demonstrated probiotic properties of lactobacilli and their effects on enhancing the quality of functional dairy foods, which gives it added importance as a potential therapeutic food component [19].

Yoghurt is considered one of the best food carriers for probiotics due to its rich nutritional composition, suitability for fermentation processes, and role in promoting digestive health. Evidence suggests that yoghurt-based diets can modify the gut microbiome and reduce the development of fatty liver disease in animal models [20,21]. Therefore, fortifying yoghurt with *Apilactopacillus kunkeei* bacteria may provide a synergistic therapeutic strategy. In addition, research on *Apilactopacillus* has revealed their ability to stimulate strong mucosal IgA responses, supporting their potential role in protecting the gut–hepatis axis [22].

Given the increasing prevalence of diet-induced fatty liver disease and the growing interest in functional foods fortified with novel probiotics, investigating the protective effects of yoghurt fortified with *Apilactopacillus kunkeei* represents an area of significant research interest. This study aims to evaluate the protective role of this functional product against fructose-induced fatty liver disease in rats by examining liver fat accumulation, metabolic function indicators, and intestinal microbiome composition.

Despite growing evidence that shows the role of probiotics in managing NAFLD, studies on investigating the efficacy of incorporating novel fructophilic probiotics, such as *Apilactopacillus kunkeei*, into functional food matrices, particularly yoghurt, and evaluating their potential effects on fructose-induced NAFLD, remain limited. Furthermore, the underlying mechanisms linking probiotic-enriched dairy products to gut microbiome modulation and hepatic lipid metabolism are not yet fully explored. Therefore, this study investigates whether yoghurt fortified with *A. kunkeei* can attenuate fructose-induced NAFLD by reducing hepatic fat accumulation and improving the gut microbiome.

## 2. Materials and Methods

### 2.1. Propagation of Apilactobacillus kunkeei DSM 12361

*A. kunkeei* DSM 12361 bacteria were imported from the German DSMZ Microorganisms and Cell Cultures GmbH (Braunschweig, Germany), cultured twice in MRS medium (Merck, Darmstadt, Germany) at pH 5.2 under anaerobic conditions using an anaerobic jar and anaerobic bag system (Oxide, Lyndhurst, Hampshire, UK), and incubated at 32 °C for 18 h.

### 2.2. Manufacture of Yoghurt

Yoghurt was prepared using YC-180 DVS culture (Chr-Hansen, Copenhagen, Denmark) according to the procedures previously described [23], whereas the pH (pH meter, Thermo Fisher Scientific, Waltham, MA, USA) of yoghurt samples ranged from 4.6 to 4.5 after four hours of incubation at 42 °C. The final viable count of yoghurt culture ranged from 8.2 to 8.4 Log CFU/g. To achieve a final viable bacterial count of *A. kunkeei* within the range of 8.00 and 8.30 Log CFU/g, yoghurt samples were supplemented with 3% active culture of *A. kunkeei*. Yoghurt cultures were counted using Lee’s agar medium (HiMedia Laboratories, Mumbai, India). *A. kunkeei* bacterial cells were counted using MRS agar medium supplemented with fructose as the sole carbon source, plus 0.05% L-cysteine hydrochloride (Merck, Darmstadt, Germany). Yoghurt culture plates were incubated at 42 °C for 48 h. *A. kunkeei* culture plates were incubated anaerobically at 37 °C for a similar period.

### 2.3. Feeding Experimental Protocol

Thirty male Wistar rats, aged 11 weeks (Wistar Han IGS, Strain code: 273 Charles Rivers, Hollister, CA, USA), weighing between 260 and 270 g, were placed individually in micro-isolator plastic cages (one rat per cage), with a basal diet and unrestricted access to water (ad libitum) for two weeks until their weights reached 340–370 g, under controlled temperature and humidity conditions and a 12 h light/dark cycle.

Rats were randomly assigned to three groups (10 rats per group) using a computer-generated random sequence (random number function in Microsoft Excel) with equal allocation. Rats in the control group (HFrD) were fed a high-fructose (70%) diet while rats in experimental groups were fed the same diet mixed with 10% of yoghurt containing YC-180 starter culture (HFrD-Y) or yoghurt containing YC-180 and *Apilactobacillus kunkeei* DSM 12361 (HFrD-Y-A). The high-fructose diet was prepared according to Kawasaki et al. [24,25]. The composition of the diet is 17% casein, 70% fructose, 3% soybean oil, 1.1% vitamin mixture, 0.3% DL-methionine, 4% mineral mixture, and 4.6% cellulose powder. Nutritional procedures, blood collection, biochemical measurements, and counting of fecal bacteria (*Enterobacteriaceae*, *lactobacilli*, and *bifidobacteria*) were performed based on the protocols of Kawasaki et al. and Elshaghabee [24,25]. All animal care and procedures were approved by Cairo University, Institutional Animal Care and Use Committee (IACUC) with approval Number: CU/I/F/29/21, 3 July 2021.

### 2.4. Blood Collection

At the end of the experiment (6 weeks), rats were anesthetized with a 4:1 ketamine-xylazine mixture at a dose of 0.25 mL/100 g body weight via intraperitoneal injection. A blood sample (1 mL) was withdrawn from a retinal vein into heparin-treated tubes. The plasma was then immediately separated by centrifugation at 4000 rpm for 10 min, and the samples were stored at −20 °C until analysis. To reduce bias, animals and all collected samples were assigned unique identification codes during laboratory measurements.

### 2.5. Determination of Biochemical Parameters in Plasma and Blood Samples

Plasma levels of alanine aminotransferase (ALT), aspartate aminotransferase (AST), triglycerides and cholesterol (Thermo Fisher Scientific, Passau, Germany), and glucose (New Blood Sugar Test, Boehringer Mannheim, Ingelheim, Germany) were measured using a spectrophotometer (SHIMADZU, Tokyo, Japan). Interleukin (IL)-6 and IL-10 levels were also estimated using commercial ELISA kits (Genzyme Diagnostics, Cambridge, UK).

### 2.6. Enumeration of Fecal Bacteria

Fecal samples were freshly collected from rats and placed directly into the anaerobic tube (Clinical Supply, Gifu, Japan) and weighed. Samples were examined for *Enterobacteriaceae*, *Lactobacilli and Bifidobacteria* according to the methods of Mitsuoka (1992) [26].

### 2.7. Determination of Fecal Ethanol

Feces samples (0.1 g from each rat) were collected and suspended in 1 mL of phosphate-buffered saline (pH 7). Fecal ethanol was determined by HPLC as described previously [27].

### 2.8. Statistical Analysis

The number of animals required for this study was estimated based on earlier experiments with a high-fructose diet [25]. No missing data were recorded, and all rats completed the study. Results were analyzed statistically with IBM SPSS statistics 25 (2017, IBM Corporation, Armonk, NY, USA). Data are expressed as mean ± standard deviation (SD). The normality of data (Shapiro–Wilk test) and homogeneity of variances (Levene’s test) were assessed. One-way analysis of variance (ANOVA) was performed at *p*-value < 0.05. Duncan’s multiple range test was used for multiple comparisons between treatments.

## 3. Results

### 3.1. Liver Enzymes and Liver Index

The results presented in Table 1 indicate that fortification of yoghurt with *A. kunkeei* reduced plasma concentrations of ALT and AST in HFrD-fed rats. Also, dietary yoghurt plus *A. kunkeei* reduced the hepatic index in HFrD-fed rats (Table 1).

### 3.2. Plasma Levels of Glucose, Triglycerides, and Total Cholesterol

The effects of adding yoghurt with the *A. kunkeei* blend on plasma concentrations of triglycerides, total cholesterol, and glucose are summarized in Table 2. The addition of yoghurt with or without *A. kunkeei* decreased plasma concentrations of triglycerides, total cholesterol, and glucose in HFrD-fed rats.

### 3.3. Fecal Bacteria

The results illustrated in Table 3 proved that supplementation of yoghurt with the *A. kunkeei* mixture reduced *Enterobacteriaceae* count, while *Lactobacilli* and *Bifidobacteria* counts were increased.

### 3.4. Fecal Ethanol

**Figure 1:**
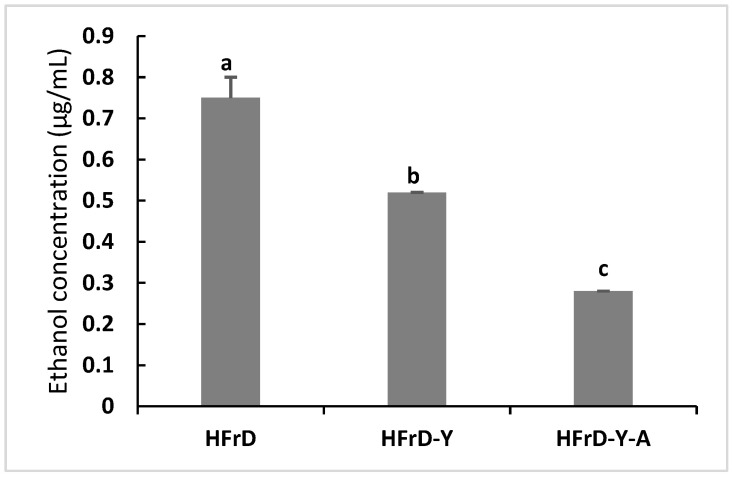
Effect of yoghurt supplemented with the *Apilactobacillus kunkeei* strain on fecal ethanol concentration (µg/mL) in rats fed a high-fructose diet. Values are means with their standard deviation depicted by vertical bars for ten rats per group. Means with different letters differ significantly (*p* < 0.05).

As graphically presented in Figure 1, fecal ethanol concentration was reduced by supplementation of dietary yoghurt alone or with *A. kunkeei* in HFrD-fed rats.

### 3.5. Plasma Levels of IL-6 and IL-10

**Figure 2:**
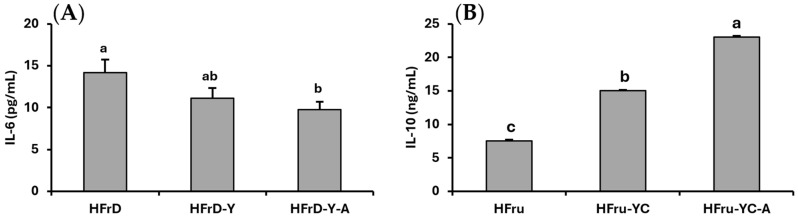
Effect of yoghurt supplemented with the *Apilactobacillus kunkeei* strain on the plasma levels of (**A**) IL-6 and (**B**) IL-10 in rats fed a high-fructose diet. Values are means with their standard deviation depicted by vertical bars for ten rats per group. Means with different letters differ significantly (*p* < 0.05).

Interestingly, the results of the present study (Figure 2) indicated that supplementation of dietary yoghurt alone or with *A. kunkeei* reduced pro-inflammatory cytokines (IL-6) and enhanced anti-inflammatory cytokines (IL-10) in HFrD-fed rats.

## 4. Discussion

### 4.1. Liver Enzymes and Liver Index

Liver function indicators like ALT and AST are important for determining the presence and severity of liver damage because high levels of such enzymes are a crucial indicator of hepatic damage. One of the most important data of the present study is that fortification of yoghurt with *A. kunkeei* reduced the plasma concentration of ALT and AST in HFrD-fed rats (Table 1). These results are consistent with earlier research showing the hepatoprotection effectiveness of probiotics [28,29,30]. Wang et al. [28] showed that supplementation of probiotic (*Lactobacillus rhamnosus* GG) culture supernatant decreased serum levels of ALT and lactate dehydrogenase (LDH) in mice. Ghosh et al. [30] noted that administration of *Lactobacillus plantarum* KAD reduced serum concentrations of ALT, AST, and gamma-glutamyl transferase (GGT) in Swiss albino mice that consumed a high-fat diet [30].

Hsieh et al. [31] postulated that *Lactobacillus plantarum* (TSP05), *Lactobacillus fermentum* (TSF331), and *Lactobacillus reuteri* (TSR332) reduced the serum concentration of ALT and AST in mice. Kong et al. elucidated that dietary yoghurt enriched with probiotics helped in managing against NAFLD (by reducing liver enzymes (ALT and AST)) as caloric dilution when incorporating a calorie-restricted diet including fruits and vegetables [20]. Moreover, Ebrahimi-Mousavi et al. concluded that the daily consumption of yoghurt enriched with probiotic reduced liver enzymes (ALT, AST, and GGT) in patients with NAFLD [32]. Regarding liver index, dietary yoghurt plus *A. kunkeei* reduced the hepatic index in HFrD-fed rats (Table 1). These results are in correspondence with Elshaghabee [25] who observed that dietary Karish cheese containing *Lactobacillus acidophilus* NRRL-B-4495, *Bifidobacterium longum* NRRL-B- 41409, and *Streptococcus thermophilus* reduced the hepatic index in rats fed HFrD-feed. Kobyliak et al. [33] documented that multi-strain probiotics decreased the fatty liver index in NAFLD patients. Similarly, Zhang et al. [34] demonstrated that *Lactobacillus casei* YRL577 significantly minimized liver weight and liver index and was involved in regulating lipid metabolism, oxidative stress, and proinflammatory cytokines in mice fed a high-fat diet. Moreover, dietary *Lactobacillus rhamnosus* B10 reduced the relative liver index via stimulating the activity of superoxide dismutase (SOD) and minimizing free radicals’ generation [28]. Thus, it might be indicated that fortification of yoghurt with *A. kunkeei* might have a positive effect on liver function indices and liver index in HFrD-fed rats. A limitation of the present study is that liver histopathology was not performed. Therefore, the severity of NAFLD could not be histologically graded.

### 4.2. Plasma Levels of Glucose, Triglycerides, and Total Cholesterol

Administration of yoghurt with or without *A. kunkeei* decreased plasma concentrations of triglycerides, total cholesterol, and glucose in HFrD-fed rats (Table 2). These findings are in alignment with several studies [16,35,36]. Xu et al. [16] postulated that dietary supplementation of *A. kunkeei* FM01 significantly reduced serum glucose levels and enhanced the serum lipid profile in the form of reducing serum total cholesterol, LDL-cholesterol, triglycerides, phosphatidylcholine, and lysophosphatidylcholine concentrations while increasing beneficial phospholipids such as phosphatidylethanolamine in HFrD-fed mice. These results are in harmony with those of Elshaghabee [25], which indicated that dietary Karish cheese containing *Lactobacillus acidophilus* NRRL-B-4495, *Bifidobacterium longum* NRRL-B- 41409, and *Streptococcus thermophilus* reduced plasma concentrations of triglycerides, cholesterol, and glucose, in rats fed HFrD-feed. At the level of gene expression, Cao et al. [29] elucidated that supplementation of *Lactobacillus plantarum* ZJUIDS14 had a positive effect on lipogenesis genes including fatty acid transport protein 2 (*fatp2*) and fatty acid transport protein 5 (*fatp5*), leading to inhibition of hepatic fat accumulation in high-fat-diet-fed mice, leading to a reduction in serum concentrations of triglycerides, total cholesterol, LDL-cholesterol, and glucose, while increasing the serum concentration of HDL-cholesterol concentrations. Likewise, Fan et al. [36] explained that supplementation of *Levilactobacillus brevis* FZU0713 enhanced the hepatic mRNA expression of acyl-CoA oxidase1 (*Acox1*) and cholesterol 7α-hydroxylase (*Cyp7a1*), which is involved in beta-oxidation of fatty acids and conversion of cholesterol to bile acids, respectively, in HFrD-fed rats. It is clear that dietary probiotic might influence hepatic lipid metabolism. Therefore, it might be speculated that dietary administration of *A. kunkeei* might help in maintaining glucose stability, improving glucose homeostasis, and stabilizing plasma lipid profile in HFrD-fed rats. These positive effects might be translated into better health in the host.

### 4.3. Fecal Bacteria

Supplementation of yoghurt with the *A. kunkeei* mixture reduced *Enterobacteriaceae* count, while *Lactobacilli* and *Bifidobacteria* counts were increased (Table 3); however, strain-specific colonization was not measured in the current study. Several previous studies pointed out that probiotics established the homeostasis of gut microbiota in hosts by inhibiting the pathogenic bacteria and stimulating the beneficial bacteria in different animal species including mice [16,29], rats [36], rabbits [37], and chickens [38,39]. Xu et al. [16] elucidated that dietary supplementation of *A. kunkeei* FM01 significantly enhanced the gut microbiome via decreasing pro-inflammatory and fructose-metabolizing species including *Alistipes*, *Oscillibacter*, *Desulfovibrio*, *Lawsonibacter*, and *Enterococcus* and increasing helpful species like *Kineothrix alysoides* and *Faecalibaculum rodentium* in HFrD-fed mice. Similarly, Vergalito et al. [17] pointed out that *A. kunkeei* had a potential inhibition of pathogenic bacteria such as *Pseudomonas aeruginosa* and *Enterococcus faecalis*, which might be involved in enhancing human health. These microbial changes were linked to improved abundances in genes encoding amino acid biosynthesis pathways and carbohydrate-active enzymes. This means that *A. kunkeei* might be involved in intestinal fructose metabolism [16]. Additionally, Cao et al. [29] postulated that high-fat-diet-fed mice that consumed *Lactobacillus plantarum* ZJUIDS14 had a re-establishment of their intestinal microflora and homeostasis including the *Coprostanoligenes* group, *Ruminococcaceae* UCG-014, *Allobaculum*, *Ruminiclostridium*, and *Roseburia*. Elshaghabee [25] observed that dietary Karish cheese containing *Lactobacillus acidophilus* NRRL-B-4 495, *Bifidobacterium longum* NRRL-B-41409, and *Streptococcus thermophilus* reduced fecal *Enterobacteriaceae* in rats fed an HFrD diet. Further studies are still needed to determine the impacts of yoghurt enriched with probiotic on strain-specific colonization and intestinal permeability markers.

### 4.4. Fecal Ethanol

Fecal ethanol concentration was reduced by dietary supplementation of yoghurt alone or with *A. kunkeei* in HFrD-fed rats (Figure 1). It is well known that ethanol production was increased in the presence of high concentrations of fructose and lactic acid bacteria [27]. Probiotics have been linked to modifying gut microbiota, enhancing antioxidative properties, lowering inflammation, and improving immune response in the host [40]. Several studies indicated that probiotics were able to mitigate the harmful effects of ethanol in particularly oxidative stress and inflammation [16,31,41]. Hsieh et al. [31] postulated that *Lactobacillus plantarum* (TSP05), *Lactobacillus fermentum* (TSF331), and *Lactobacillus reuteri* (TSR332) reduced ethanol-induced liver oxidative stress and pro-inflammatory cytokines (TNF-α and IL-6) in mice. Forsyth et al. [42] showed that *Lactobacillus rhamnosus* GG supplementation reduced gut oxidative stress, enhanced ethanol-induced gut leakiness, and minimizing inflammation in rats.

### 4.5. Plasma Levels of IL-6 and IL-10

One of the most important results of the present study is that dietary administration of yoghurt alone or with *A. kunkeei* reduced pro-inflammatory cytokine (IL-6) and enhanced anti-inflammatory cytokine (IL-10) in HFrD-fed rats. These results are in agreement with Xu et al. [16] who postulated that dietary supplementation of *A. kunkeei* FM01 significantly reduced serum concentrations of pro-inflammatory cytokines IL-6 and tumour necrosis factor-α (TNF-α) in HFrD-fed mice. Ghosh et al. [30] noted that administration of *Lactobacillus plantarum* KAD exerted an anti-inflammatory effect in the form of decreasing levels of IL-6 and TNF-α, as well as showed an antioxidative effect in the form of enhancing concentrations of SOD, glutathione (GSH), and catalase (CAT) and reducing malondialdehyde (MDA) concentration in the serum, liver, and colon in Swiss albino mice consuming a high-fat diet. Also, Korkmaz et al. [41] reported that supplementation of *Lactobacillus Plantarum* and *Lactobacillus Helveticus* decreased the renal concentrations of cytokines (TNF-α, IL-1β, IL-6, and IL-10) in HFrD-fed rats. Elshaghabee [25] observed that dietary Karish cheese containing *Lactobacillus acidophilus* NRRL-B-4495, *Bifidobacterium longum* NRRL-B-41409, and *Streptococcus thermophilus* reduced plasma concentrations of IL-6 and increased plasma concentrations of IL-10 in rats fed HFrD. Similarly, Poutahidis et al. [43] observed that consumption of yoghurt containing *Limosilactobacillus reuteri* reduced the concentration of IL-6 and improved the concentration of IL-10. Taken together, these findings showed that *A. kunkeei* defends against HFrD-induced inflammation, supporting its role in enhancing gut health in the perspective of high-fructose intake.

While several studies showed that probiotics were significantly able to reduce liver enzymes like ALT and AST in conditions like NAFLD, other studies found no significant effect [29,44,45]. The conflicting findings in the NAFLD-probiotic field could be summarized as follows: (1) the results of meta-analyses differ on whether probiotics enhance BMI [44], (2) the probiotic intervention is heterogeneous [45], and (3) the data of clinical studies on the gut microbiome are heterogeneous and often conflicting [29]. This conflict might be related to probiotic strain, duration, dosage, patient population (obese vs. non-obese), and study design, highlighting the need for more specific research on effective strains and treatments for liver health.

## 5. Conclusions

In summary, excessive fructose intake is a risk factor that is linked to NAFLD. One possible mechanism is the fermentation of fructose by gut microbiota, which resulted in a subsequent increase in endogenous ethanol that promotes hepatic fat accumulation. In this context, we developed a functional yoghurt fortified with *Apilactobacillus kunkeei* DSM 12361 and studied its effect in rats fed a high-fructose diet. Fortification of yoghurt with *A. kunkeei* improved liver function by reducing plasma levels of ALT and AST and a lower liver index. These benefits were accompanied by an improved blood lipid profile and a beneficial change in the gut microbiota, shown by decreased *Enterobacteriaceae* and increased *Lactobacilli* and *Bifidobacteria* counts. This microbial change was associated with reduced fecal ethanol levels, reduction in the pro-inflammatory cytokine IL-6, and improvement in the anti-inflammatory cytokine IL-10. However, this study is limited by the absence of liver histopathology for NAFLD grading, the lack of oxidative stress markers, and mechanistic gene-expression analyses. Therefore, further studies are suggested to (i) confirm hepatic outcomes using histological scoring, (ii) expand mechanistic characterization using oxidative stress indices and targeted gene-expression profiling, and (iii) determine the effects of probiotic-enriched yoghurt on strain-specific colonization and intestinal barrier function.

## Figures and Tables

**Table 1:** Effect of yoghurt supplemented with *Apilactobacillus kunkeei* strain on plasma liver enzymes (mg/mL) and liver index (%) in rats fed a high-fructose diet.

Group	ALT	AST	Hepatic Index
HFrD	52.52 ± 3.2 ^a^	82.35 ± 6.1 ^a^	5.12 ± 0.8 ^a^
HFrD-Y	42.58 ± 3.5 ^ab^	67.51 ± 5.8 ^b^	4.1 ± 0.5 ^ab^
HFrD-Y-A	26.6 ± 3.1 ^c^	54.35 ± 5.4 ^bc^	2.63 ± 0.4 ^c^

HFrD; high-fructose diet, HFrD-Y; high fructose diet + yoghurt, HFrD-Y-A; high fructose + yoghurt + *Apilactobacillus kunkeei*, ALT; alanine transaminase, AST; aspartate aminotransferase. Values are means ± standard deviation for ten rats per group. Means with distinct superscript letters (a, b, c) denote significant differences among groups (*p* < 0.05).

**Table 2:** Effect of yoghurt supplemented with the *Apilactobacillus kunkeei* strain on plasma levels of glucose, triglycerides, and total cholesterol in rats fed a high-fructose diet.

Group	Triglycerides	Total Cholesterol	Glucose
HFrD	370.15 ± 28 ^a^	160.25 ± 11 ^a^	375.25 ± 11 ^a^
HFrD-Y	299.3 ± 17 ^b^	140.52 ± 8.1 ^b^	299.62 ± 15 ^b^
HFrD-Y-A	225.15 ± 13 ^c^	103.31 ± 9.5 ^c^	230.15 ± 10 ^c^

HFrD; high-fructose diet, HFrD-Y; high-fructose diet + yoghurt, HFrD-Y-A; high fructose + yoghurt + *Apilactobacillus kunkeei*. Values are means ± standard deviation for ten rats per group. Means with distinct superscript letters (a, b, c) denote significant differences among groups (*p* < 0.05).

**Table 3:** Effect of yoghurt supplemented with the *Apilactobacillus kunkeei* strain on the viable count (Log CFU/g) of fecal bacteria in rats fed a high-fructose diet.

Group	Fecal *Enterobacteriaceae*	Fecal *Lactobacilli*	Fecal *Bifidobacteria*
HFrD	6.35 ± 1.05 ^a^	5.23 ± 0.81 ^b^	4.05 ± 0.51 ^b^
HFrD-Y	5.12 ± 0.72 ^b^	6.15 ± 0.53 ^a^	4.75 ± 0.63 ^a^
HFrD-Y-A	3.52 ± 1.32 ^b^	6.80 ± 0.65 ^a^	5.45 ± 0.52 ^a^

HFrD; high-fructose diet, HFrD-Y; high-fructose diet + yoghurt, HFrD-Y-A; high fructose + yoghurt + *Apilactobacillus kunkeei*. Values are means ± standard deviation for ten rats per group. Means with distinct superscript letters (a, b) denote significant differences among groups (*p* < 0.05).

## Data Availability

The original contributions presented in this study are included in the article. Further inquiries can be directed to the corresponding author.
